# Nanocarrier-Mediated Non-Invasive Drug Delivery for Wet Age-Related Macular Degeneration: Advances and Translational Challenges

**DOI:** 10.3390/pharmaceutics18070861

**Published:** 2026-07-15

**Authors:** Shasha Wang, Linfei Liu, Xiaoling Zeng, Chonghui Tang, Wei Chen, Xuri Li, Weisi Lu

**Affiliations:** State Key Laboratory of Ophthalmology, Zhongshan Ophthalmic Center, Sun Yat-Sen University, Guangdong Provincial Key Laboratory of Ophthalmology and Visual Science, Guangzhou 510060, China

**Keywords:** wet age-related macular degeneration, nanocarriers, non-invasive ocular delivery, translational challenges

## Abstract

Wet age-related macular degeneration (wAMD) is characterized by choroidal neovascularization (CNV) and remains a major cause of severe vision loss in older adults. Intravitreal anti-vascular endothelial growth factor (anti-VEGF) therapy is the current standard of care for wAMD. However, repeated injections are associated with poor adherence, procedure-related complications, and a substantial cumulative treatment burden. Topical nanocarrier-based systems have therefore attracted increasing attention as needle-free approaches for improving posterior segment drug exposure. Complementing broader reviews of ocular nanomedicine, this review specifically examines topical nanocarrier-mediated posterior segment delivery for wAMD, with a focus on three representative platforms: liposomes, polymeric nanoparticles, and polymeric micelles. These systems are engineered through the optimization of particle size, surface properties, drug-loading strategies, and functional modifications to improve payload stability, ocular surface residence, tissue penetration, and lesion-relevant delivery. By integrating formulation design, ocular barrier transport, ocular posterior segment bioavailability, and translational feasibility in the context of wAMD, this review provides a disease-focused and application-oriented perspective that complements existing broader reviews of ocular nanocarriers and ophthalmic nanomedicine. We summarize current evidence from preclinical and translational studies and discuss major barriers limiting clinical application, including insufficient posterior segment drug exposure, dose–safety trade-offs, pharmacokinetic instability, limited targeting efficiency, and challenges in delivering macromolecular biologics, such as anti-VEGF antibodies and fusion proteins. At present, topical nanocarrier-based strategies remain investigational, but they hold potential for development as therapeutic approaches for wAMD. Key priorities for future development include quantitative posterior segment pharmacokinetic/pharmacodynamic evaluation, long-term safety assessment, payload-specific carrier design, scalable manufacturing, and clinically relevant efficacy endpoints. This review provides a focused framework for the rational design and translational assessment of nanocarrier-based topical strategies for wAMD management.

## 1. Introduction

Age-related macular degeneration (AMD) is a leading cause of irreversible visual impairment in older adults [1]. With population ageing, the global burden of AMD continues to increase, and the number of affected individuals is projected to reach approximately 288 million by 2040 [1]. It is broadly classified into dry AMD and wet AMD (wAMD) [2]. Although wAMD accounts for only about 10–15% of AMD patients, it is responsible for the majority of cases of severe vision loss [3,4]. wAMD is characterized by choroidal neovascularization (CNV) and associated manifestations such as retinal pigment epithelial (RPE) detachment, subretinal hemorrhages, and fibrovascular disciform scarring, which ultimately lead to visual decline or irreversible vision loss [5].

Anti-vascular endothelial growth factor (anti-VEGF) therapy is the first-line treatment for wAMD [6,7]. Clinically used intravitreal agents include ranibizumab, aflibercept, brolucizumab, and faricimab [8,9,10,11,12]. Among these agents, faricimab blocks both VEGF-A and angiopoietin-2 [12]. In addition, VEGF-C/D signaling has been explored as a potential therapeutic target in wAMD [13]. However, these therapies rely heavily on invasive intravitreal injections, which carry risks such as infection, intraocular hemorrhage, retinal detachment, and poor patient compliance [14,15,16,17]. Therefore, the development of safer, more patient-friendly, and sustainable therapeutic strategies is of considerable clinical importance.

In recent years, topical ocular drug delivery has emerged as an important research direction in wAMD therapy. In this review, we focus on topical administration as the main non-invasive route, as it avoids needle-based ocular procedures and is more compatible with long-term patient-centered treatment. Other delivery approaches that require procedural intervention, such as periocular, suprachoroidal, or implant-based delivery, are only briefly discussed to provide context for posterior segment drug delivery and are beyond the primary scope of this review [18]. However, non-invasive delivery, especially to the posterior segment, remains challenged by multiple ocular barriers, including the corneal barrier, the blood–aqueous barrier, and the blood–retinal barrier. These barriers markedly restrict effective drug exposure in the retina and choroid [19].

Nanocarriers have attracted increasing attention as potential platforms for non-invasive ocular delivery because of their capacity to improve drug solubility, prolong ocular surface residence, and enhance penetration [20,21,22]. In this review, we outline the major routes of non-invasive ocular drug delivery and summarize recent advances in topical nanocarrier-mediated posterior segment delivery. Given the focus on wAMD, this review intentionally highlights liposomes, polymeric nanoparticles, and polymeric micelles as representative nanocarrier platforms. These systems encompass lipid-based, polymer-based, and self-assembled architectures and have been the most extensively investigated for topical ocular drug delivery. Focusing on these representative platforms enables a structured comparison of formulation design, ocular barrier transport, posterior segment drug exposure, and translational potential. We further discuss the key issues limiting their clinical translation in wAMD management and highlight future directions for rational optimization.

## 2. Literature Search and Selection

This narrative review was based on a targeted literature search conducted in PubMed for studies published up to 4 June 2026. Search terms included combinations of “wet age-related macular degeneration,” “posterior segment drug delivery,” “topical ocular delivery,” “nanocarriers,” “liposomes,” “polymeric nanoparticles,” and “polymeric micelles.” Preclinical investigations, review articles, and publications addressing translational and regulatory considerations were screened. Studies were selected based on their relevance to topical nanocarrier-mediated posterior segment drug delivery for wAMD, with a particular focus on liposomes, polymeric nanoparticles, and polymeric micelles, because these represent the most extensively investigated platforms, providing sufficient evidence to support meaningful comparisons of ocular transport and translational potential.

## 3. Major Routes of Non-Invasive Ocular Delivery

Intravitreal injection (IVT) is the standard route for anti-VEGF therapy in wAMD. However, repeated IVT administration imposes substantial temporal, financial, and psychological demands on patients [23] and increases the cumulative risk of severe ocular complications, including endophthalmitis and retinal detachment [14]. Consequently, non-invasive and patient-friendly ocular drug delivery strategies, particularly topical eye-drop formulations, have emerged as a major research focus for reducing dependence on repeated IVT injections [24]. Accordingly, throughout this review, “non-invasive ocular delivery” primarily refers to topical eye-drop administration that avoids needle penetration, implantation, or disruption of ocular tissues. Other procedural or systemic routes, including intravenous verteporfin followed by laser activation, are discussed only for contextual comparison.

**Figure 1:**
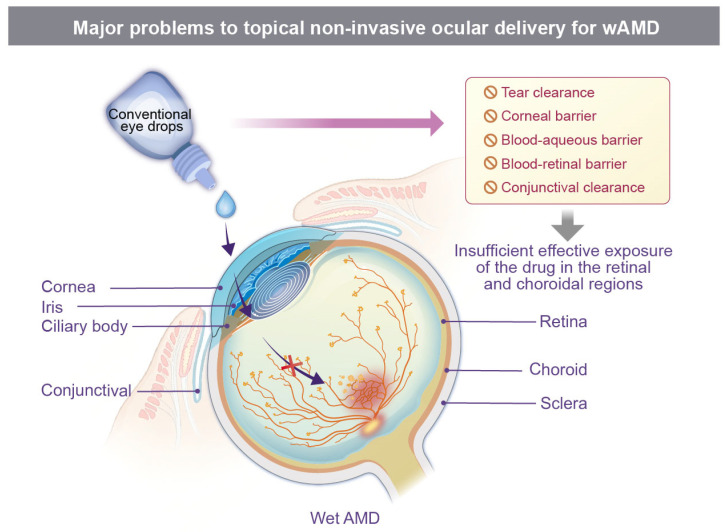
Major barriers to topical non-invasive ocular delivery for wet age-related macular degeneration (wAMD). Conventional eye drops are rapidly cleared from the ocular surface and encounter multiple anatomical and physiological barriers, including tear and conjunctival clearance, the corneal barrier, the blood–aqueous barrier, and the blood–retinal barrier. These barriers limit effective drug exposure in posterior segment tissues, including the retina, the RPE–choroid complex, and choroidal neovascularization lesions.

Effective posterior segment delivery via conventional topical administration remains challenging because of multiple physiological barriers, including the corneal epithelial barrier, conjunctival clearance mechanisms, scleral stromal resistance, and the blood–retinal barrier (Figure 1) [25]. Consequently, conventional eye drops rarely achieve sustained and therapeutically effective drug exposure in the posterior segment. Therefore, improving transport across ocular barriers and enhancing posterior ocular bioavailability have become central challenges in the development of non-invasive therapeutic strategies.

In recent years, small-molecule tyrosine kinase inhibitors (TKIs) have attracted increasing attention as potential candidates under investigation for topical wAMD therapy because of their relatively low molecular weight and favorable tissue permeability. For instance, a preclinical study demonstrated that an SAC4A/sunitinib eye-drop formulation based on host–guest supramolecular chemistry markedly enhanced the posterior segment bioavailability of sunitinib. In this system, SAC4A functions as a host molecule that efficiently encapsulates sunitinib via non-covalent interactions and enables stable drug delivery. Sunitinib, a multitarget TKI, primarily suppresses VEGFR- and PDGFR-mediated signaling pathways. In a laser-induced CNV mouse model, this topical formulation significantly inhibited neovascularization, indicating anti-angiogenic activity in this preclinical model [26]. In parallel, PAN-90806, a topical VEGFR2-targeting inhibitor, was evaluated in a Phase I/II clinical trial involving 51 treatment-naïve patients with wAMD (NCT03479372).

However, the clinical translation of topical TKIs remains difficult. In a Phase II clinical trial involving 90 previously treated patients with wAMD, topical acrizanib (LHA510), a VEGFR2/KDR-targeting small-molecule inhibitor, failed to reduce patients’ reliance on rescue intravitreal anti-VEGF injections and was associated with reversible corneal haze and other ocular adverse events [27]. Similarly, topical regorafenib, another multikinase inhibitor investigated for wAMD, was evaluated in a Phase IIa/IIb clinical program that was terminated after Phase IIa because its efficacy was lower than that of current wAMD treatments [28]. These findings indicate that the theoretical permeability advantages of small-molecule TKIs do not necessarily translate into stable and therapeutically sufficient posterior segment drug concentrations and may also raise ocular safety concerns.

Beyond small-molecule therapeutics, the non-invasive delivery of macromolecular anti-VEGF agents remains largely at the preclinical stage and mainly relies on nanocarrier-based delivery systems, as discussed in detail below.

## 4. Advances in Nanocarriers for Non-Invasive Ocular Delivery in wAMD

**Figure 2:**
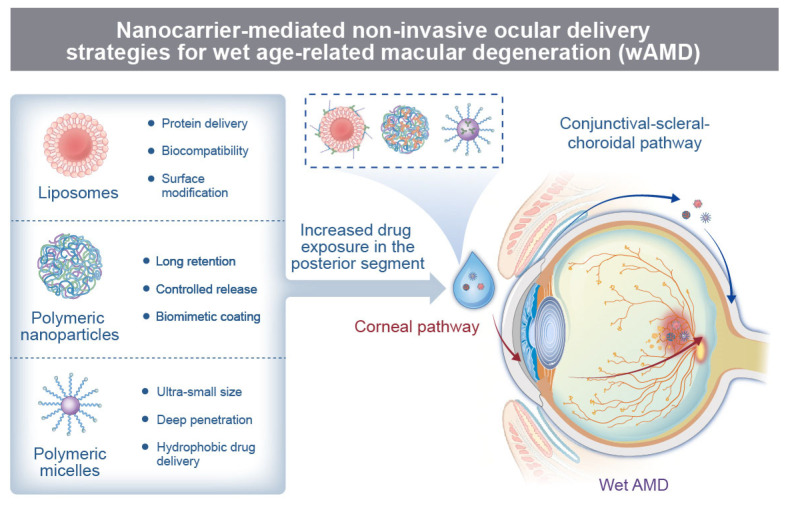
Nanocarrier-mediated topical ocular delivery strategies, therapeutic effects, and translational challenges for wAMD. Liposomes, polymeric nanoparticles, and polymeric micelles can improve drug stability, ocular retention, tissue penetration, controlled release, and the solubilization of hydrophobic drugs. Posterior segment delivery is proposed to occur mainly through the conjunctival–scleral–choroidal pathway, with the corneal pathway serving as a secondary route. These platforms may provide anti-angiogenic effects, reduce vascular leakage, sustain drug release, and improve bioavailability. However, clinical translation remains constrained by insufficient posterior segment exposure, ocular toxicity, pharmacokinetic uncertainty, limited protein delivery, and manufacturing challenges.

Nanocarrier-based delivery systems have attracted considerable attention in recent years for overcoming the limited posterior segment delivery efficiency, insufficient bioavailability, and short ocular residence time associated with conventional eye drops [20]. Nanocarriers are nanoscale delivery platforms capable of transporting therapeutic agents through encapsulation, adsorption, or conjugation while regulating intraocular drug transport through material composition, particle size, surface charge, surface functionalization, and release kinetics [29]. Common ophthalmic nanocarriers include liposomes, polymeric nanoparticles, polymeric micelles, dendrimers, solid lipid nanoparticles, nanoemulsions, and nanogels. Accordingly, liposomes, polymeric nanoparticles, and polymeric micelles are discussed here as representative nanocarriers for non-invasive ocular drug delivery in wAMD (Figure 2; Table 1), with emphasis on their structural characteristics, delivery advantages, and recent wAMD-related progress.

Liposomes are classic nanodelivery systems composed of phospholipid bilayers. Because of their hydrophilic aqueous core and hydrophobic lipid bilayer, they can encapsulate both water-soluble macromolecules, such as anti-VEGF proteins and antibodies, and small hydrophobic molecules, including TKIs [30]. In the context of non-invasive therapy for wAMD, the principal advantages of liposomes lie in their favorable biocompatibility, dual-phase drug-loading capacity, and surface modifiability. Functional ligands, such as cell-penetrating peptides and HA, can be introduced onto the liposomal surface to enhance ocular surface adhesion, trans-tissue penetration, and retina-targeted accumulation [31]. In addition, liposomal encapsulation may reduce the risk of enzymatic degradation and inactivation of therapeutic agents while prolonging ocular residence time.

In a laser-induced CNV mouse model, topical administration of conbercept-loaded liposomes dual-modified with Penetratin and HA (PenHA-Lip/Conb) increased posterior segment bioavailability and effectively suppressed CNV formation and vascular leakage [31]. Among clinically applied liposomal formulations, verteporfin liposomes (Visudyne^®^) have been used for photodynamic therapy in patients with AMD, demonstrating the feasibility of liposome-based approaches for CNV treatment [32]. However, Visudyne^®^ should not be considered an example of non-invasive or topical ocular delivery, because it requires intravenous infusion followed by laser activation and differs fundamentally from eye-drop-based posterior segment delivery. Therefore, although Visudyne^®^ provides a useful clinical reference for liposome-based therapy in AMD, direct evidence supporting liposomal eye drops as an alternative to intravitreal injection remains lacking (NDA 21-119/S-001, FDA).

Overall, liposomes offer favorable biocompatibility, dual-phase drug-loading capacity, and controlled-release properties, making them an important platform for the development of non-invasive therapeutic strategies for wAMD (Table 1).

Polymeric nanoparticles (PNPs), fabricated from polymers such as PLGA, PLA, PCL, and chitosan, represent a versatile class of nanocarriers that can achieve stable drug delivery through drug encapsulation, surface adsorption, or functional modification [33,34,35]. PNP-based drug delivery represents a promising strategy for wAMD treatment, owing to its capacity to prolong drug retention within the retina–choroid complex and CNV lesions.

Studies employing PNPs as non-invasive topical eye drops for wAMD therapy are limited. A representative example was reported by Chu et al., who developed iRGD/TAT dual-functionalized PEG–PLGA nanoparticles for ocular surface delivery to laser-induced CNV lesions. TAT enhanced penetration across ocular-surface barriers, whereas iRGD promoted CNV-directed accumulation by interacting with integrin αvβ3 expressed on neovascular endothelial cells. Following topical instillation, these dual-modified nanoparticles were able to reach laser-induced CNV lesions, providing proof-of-concept evidence that PNPs can access wAMD-related posterior segment lesions [36]. However, as this study primarily demonstrated high corneal permeability and posterior segment delivery rather than definitive anti-CNV efficacy, it should be interpreted as evidence supporting PNPs as a non-invasive ocular surface delivery platform rather than proof of therapeutic efficacy in wAMD.

To date, PNPs have been more extensively studied for wAMD therapy through non-intravitreal administration routes. For example, Angiopoietin-1/anti-CD105-conjugated PLGA nanoparticles administered by tail-vein injection were designed to recognize CNV neovascular endothelium through anti-CD105-mediated targeting, while sustained release of Angiopoietin-1 helped stabilize the vascular barrier. In a laser-induced CNV model, this strategy significantly reduced vascular leakage and CNV lesion area [33].

Although topical PNP-based therapy for wAMD remains underexplored, accumulating evidence suggests that PNP eye drops can reach posterior ocular tissues and may be applicable to posterior segment diseases. For instance, surface-modified PLGA nanoparticles administered as eye drops were detected in the mouse retina, with fluorescence-labeled nanoparticles detectable in retinal tissue. Compared with unmodified PLGA nanoparticles, chitosan-modified nanoparticles exhibited enhanced retinal delivery, supporting the feasibility of PNP-mediated posterior segment delivery after topical administration in mice [37]. In another study, SA-2-loaded PLGA nanosuspension eye drops achieved ocular bioavailability in both anterior and posterior ocular tissues, produced sustained intraocular pressure reduction, and preserved retinal ganglion cell function in a mouse microbead-occlusion model of ocular hypertension [38]. Collectively, these findings suggest that appropriately engineered PNPs can improve ocular residence, enable sustained drug release, and facilitate drug access to posterior ocular tissues after topical administration, which remains difficult to achieve with conventional eye drops.

In summary, available evidence supports the feasibility of topical PNP administration for ocular disease treatment, particularly for enhancing posterior segment access and facilitating the delivery of poorly permeable therapeutic agents (Table 1).

Polymeric micelles are nanoscale colloidal carriers formed by the self-assembly of amphiphilic molecules in aqueous media and have a hydrophobic core and a hydrophilic shell [39,40]. Compared with liposomes and polymeric nanoparticles, polymeric micelles offer advantages such as ultra-small size, superior solubilization capacity for hydrophobic drugs, and the capacity to facilitate ocular tissue penetration [17,39]. Their hydrophilic shell may also reduce clearance through tears and blinking, prolong ocular surface retention, and improve bioavailability [41].

Research investigating polymeric micelles as non-invasive topical formulations for wAMD therapy remains extremely limited. A representative preclinical example was reported by Zhao et al., who developed a nanomicellar delivery system based on copolymer EPC (nEPCs), composed of poly(ethylene glycol), poly(propylene glycol), and polycaprolactone segments, for the encapsulation and delivery of aflibercept. In a laser-induced CNV mouse model, topically administered nEPCs facilitated the transport of aflibercept to the posterior segment. Notably, the vitreous concentration of aflibercept achieved with nEPC-encapsulated aflibercept was approximately fourfold higher than that obtained with topically administered free aflibercept. In addition to improving aflibercept delivery, nEPCs were reported to exhibit intrinsic antiangiogenic activity. The combination of improved posterior segment delivery of anti-VEGF agents and the inherent antiangiogenic properties of the nanomicelles may confer synergistic therapeutic benefits, highlighting the potential of this platform for non-invasive treatment of wAMD. However, these effects have thus far been demonstrated only in a preclinical CNV model and require further validation before their therapeutic relevance to wAMD can be established [42].

Although the direct application of polymeric micelles for wAMD therapy remains limited, existing preclinical evidence suggests that polymeric micelle-based eye-drop formulations can enhance drug delivery to posterior ocular tissues compared with conventional eye drops. For instance, one study used ultrasmall polymeric micelles encapsulating bevacizumab (P@Beva) for non-invasive topical delivery to posterior eye tissues. In ex vivo tissue-transport experiments, P@Beva enhanced transport across the cornea and the conjunctival–scleral–choroidal pathway by 23-fold and 7.9-fold, respectively [43]. Xu et al. developed chitosan oligosaccharide–valylvaline–stearic acid (CSO-VV-SA) polymeric micelles for dexamethasone delivery. Fluorescence imaging of the sclera–choroid–retina complex in rats revealed that the nanomicelles modified drug distribution within ocular tissues and enabled delivery to the posterior segment. These results provide proof-of-concept evidence that CSO-VV-SA nanomicelles may serve as a viable vehicle for non-invasive posterior ocular drug delivery [44].

In summary, the small particle size and tissue-penetration capacity of polymeric micelles support their continued investigation as topical ocular drug delivery carriers (Table 1).

Taken together, liposomes, polymeric nanoparticles, and polymeric micelles show promise for topical delivery to the posterior segment. However, clinical evidence supporting their use in wAMD remains scarce and largely preclinical. Further studies are needed to confirm posterior segment PK/PD, disease-specific efficacy, safety, formulation reproducibility, and clinically meaningful benefits.

**Table 1:** Representative nanocarrier-based drug delivery systems for wAMD therapy.

Nanocarrier	Representative Formulation	Particle Size	Zeta Potential	Surface Modification	Payload	Administration Route	Key Delivery Findings	Therapeutic Efficacy	Translational Status
Liposome	Penetratin peptide and hyaluronic acid dual-modified phospholipid liposome; HA surface coating with cell-penetrating peptide modification	152.4 ± 1.3 nm	−4.3 ± 0.9 mV	Penetratin for ocular penetration; HA for ocular surface retention/RPE targeting	Conbercept (~143 kDa)	Eye drops	Enhanced ocular penetration, surface retention, and posterior segment delivery [31].	In a laser-induced CNV mouse model, PenHA-Lip/Conb suppressed CNV formation and vascular leakage [31].	Preclinical
	Verteporfin-loaded phospholipid liposomal formulation (commercial liposomal photosensitizer, Visudyne^®^)	150–300 nm	Not reported	No active targeting ligand; conventional DMPC/egg phosphatidylglycerol liposome	Verteporfin (~719 Da small-molecule photosensitizer)	Intravenous infusion + laser activation (not non-invasive)	Enabled systemic delivery and light-triggered photodynamic activation [32].	Clinically used for AMD-related CNV; however, its effect relies on photodynamic vascular occlusion rather than anti-VEGF delivery [32].	Approved/clinical
Polymeric nanoparticles	mPEG-PLGA/iRGD-PEG-PLGA/TAT-PEG-PLGA; nanoprecipitation	67.0 ± 1.7 nm	−6.63 ± 0.43 mV	iRGD + TAT dual peptide modification; ~80% peptide conjugation to PEG–PLGA	Nile red and coumarin-6 (~670 Da)	Eye drops	Enhanced penetration promoted CNV-directed accumulation reached laser-induced CNV lesions [36].	not definitive anti-CNV therapeutic efficacy [36].	Preclinical
	Ang1-loaded PLGA-COOH NPs prepared by W/O/W double emulsion; anti-CD105 conjugated by EDC/NHS to form AAP NPs	213 ± 17.7 nm (AAP NPs)	−33.81 ± 3.57 mV (AAP NPs)	Anti-CD105 antibody on PLGA surface; antibody coupling rate 98.3%	Angiopoietin-1 (~70 kDa)	Tail-vein injection (not non-invasive)	Targeted delivery to CNV with sustained vascular barrier stabilization [33].	Significantly reduced vascular leakage and CNV lesion area in laser-induced CNV models from day 7 through day 28 post-laser induction [33].	Preclinical
	Coumarin-6-loaded PLGA NPs prepared by emulsion solvent diffusion (ESD) with PVA; surface-modified using CS, GCS, or P80	220–590 nm	−41.3 ± 9.5 mV to 39.9 ± 4.2 mV	Surface adsorption/modification with CS, GCS, or P80 to improve ocular tissue interaction	Coumarin-6 (~350 Da)	Eye drops	Enhanced retinal delivery compared with unmodified PLGA nanoparticles [37].	Not mentioned.	Preclinical
Polymeric Micelles	EPC copolymer (PEG-PPG-PCL), 2 wt% concentration, encapsulation efficiency ≈ 47.3%, hydrodynamic size ≈ 64.5 nm.	~64.5 nm	Not reported	Not reported	Aflibercept (~115 kDa)	Eye drops	Vitreous aflibercept concentration approximately 4-fold higher than free drug; micelles showed intrinsic antiangiogenic activity [42].	In laser-induced CNV models, fluorescein leakage area reduced most effectively (recovery rate 568.1 ± 68.9 pixels/day), superior to aflibercept alone or nEPCs alone [42].	Preclinical
	Amphiphilic copolymer of poly(ethylene glycol) methyl ether methacrylate (mPEG-MMA) and decyl methacrylate (DEC-MMA) at 1:3 molar ratio, Mn ≈ 32.1 kDa.	~11.57 nm	Near-neutral (≈0 mV)	Not reported	Bevacizumab (~150 kDa)	Eye drops	Ex vivo corneal transport improved 23-fold; conjunctival–scleral–choroidal transport improved 7.9-fold [43].	Efficacy inferred from achieving therapeutic concentrations [43].	Preclinical
	CSO-VV-SA, with VV:CSO-SA weight ratio 5:4, size ≈ 100 nm (TEM).	~100 nm	>+30 mV	Valylvaline (VV)—PepT-1 targeting ligand	Dexamethasone (~392 Da)	Eye drops	Modified drug distribution; fluorescence imaging confirmed delivery to posterior segment [44].	Not tested in disease models; efficacy inferred from achieving therapeutic concentrations [44].	Preclinical

## 5. Translational Challenges of Nanocarrier-Mediated Non-Invasive Ocular Delivery

Nanocarrier-mediated non-invasive ocular delivery is being investigated as a potential strategy for wAMD and could help reduce the burden of repeated intravitreal injections, improve patient adherence, and support more sustained drug exposure. However, despite encouraging preclinical progress, clinical translation remains limited, revealing a persistent gap between formulation innovation and therapeutic implementation (Figure 2) [19,45].

Achieving adequate and sustained drug exposure in the posterior segment remains a major challenge. After topical or other non-invasive administration, nanocarriers must first overcome rapid ocular surface clearance, such as tear turnover and blinking, before traversing multiple anatomical and physiological barriers to reach the retina and choroid. These clearance mechanisms markedly shorten the residence time of nanocarriers within ocular tissues and reduce their effective local concentration, thereby compromising carrier stability, drug-release kinetics, and posterior segment bioavailability [39]. Although strategies such as enhanced mucoadhesion, optimization of particle size and surface charge, and functional surface engineering may improve tissue penetration and transport across ocular barriers, these modifications may also introduce additional concerns, including ocular surface irritation, transient visual disturbance, and long-term tissue accumulation [46]. Thus, improving delivery efficiency must be carefully balanced against local tolerability and long-term ocular safety.

Safety assessment and intraocular pharmacokinetics represent another major translational bottleneck. Most current in vivo studies rely on rodent or rabbit models, yet these species differ substantially from humans in ocular anatomy, tear turnover, blinking frequency, vitreous volume, and transporter expression profiles. These interspecies differences limit the ability of conventional preclinical pharmacokinetic studies to predict human drug exposure, therapeutic durability, and potential toxicity [47]. Moreover, the deep intraocular distribution, metabolic fate, clearance pathways, and long-term retention of nanoparticles remain difficult to monitor dynamically in vivo [17]. In this context, future translational studies should incorporate more clinically relevant models, particularly non-human primates, together with high-sensitivity non-invasive imaging, time-resolved tracing approaches, and pharmacokinetic/pharmacodynamic modeling. Such integrated strategies may provide more reliable guidance for dose optimization, exposure–response assessment, and long-term safety evaluation.

The non-invasive delivery of macromolecular biologics presents an additional layer of complexity. Many polymeric micelles and related nanocarriers are intrinsically better suited for hydrophobic small-molecule drugs than for high-molecular-weight, water-soluble protein therapeutics. For anti-VEGF biologics, encapsulation efficiency, limited drug loading, and potential disruption of protein conformational stability remain key formulation constraints [48,49]. Even when nanocarriers successfully cross the ocular surface or corneoscleral barriers, their ability to selectively accumulate in the retina, retinal pigment epithelium, and choroidal neovascular lesions remains modest. In addition, the small fraction of particles that reaches the posterior segment may be sequestered by non-target tissues or rapidly eliminated through the choroidal circulation [50,51]. Therefore, improving lesion-specific enrichment and enabling spatially and temporally controlled drug release will be essential for advancing non-invasive anti-VEGF delivery from experimental proof-of-concept toward clinical application.

To address these limitations, recent efforts have focused on more sophisticated platform designs, including nanoparticle–in situ gel composite systems, microenvironment-responsive materials, biomimetic stealth coatings, highly hydrated nanogels, and porous carriers. These systems are designed to improve drug stability, prolong ocular retention, and enhance posterior segment delivery. For example, mesoporous silica nanoparticles loaded with anti-VEGF agents and incorporated into injectable hydrogels have been reported to extend drug retention at the vitreoretinal interface and improve sustained-release performance in preclinical settings [52]. Although injectable systems are beyond the scope of non-invasive ocular drug delivery, they provide useful insights for the future development of sustained-release strategies. Cequa^®^, a 0.09% cyclosporine nanomicellar ophthalmic solution, demonstrates that nanomicellar formulations can achieve clinical translation in ophthalmology. However, it is an anterior-segment product and does not establish the feasibility or efficacy of nanocarrier-mediated posterior segment delivery for wAMD [53].

Overall, the clinical translation of nanocarrier-mediated non-invasive therapies for posterior segment diseases such as wAMD requires more than formulation optimization alone. Successful posterior segment delivery should not be defined solely by drug detectability in posterior ocular tissues, but by clinically meaningful pharmacokinetic and pharmacodynamic (PK/PD) performance. Specifically, an effective delivery system should achieve quantifiable and reproducible exposure of the active therapeutic agent in disease-relevant posterior tissues, such as the retina, RPE, choroid, and/or vitreous, while limiting excessive anterior segment and systemic exposure. This target-tissue exposure should be maintained above a relevant pharmacological threshold for a clinically meaningful duration and should be linked to disease-relevant PD outcomes, including VEGF pathway suppression, inhibition of choroidal neovascularization, reduction of vascular leakage, improvement in retinal structure, or preservation of visual function-related endpoints. Future development should establish an integrated evidence framework encompassing material biocompatibility, lesion-targeting specificity, target-tissue PK/PD evaluation in human-relevant large-eye models, exposure response linked efficacy, long-term ocular and systemic safety, scalable manufacturing, batch-to-batch reproducibility, formulation stability, and clinically acceptable quality-control standards. Addressing these challenges will be essential for transforming nanocarrier-based non-invasive ocular delivery systems into safe, durable, and patient-friendly therapeutic platforms.

## 6. Future Perspectives and Optimization Strategies for wAMD Nanocarrier Translation

### 6.1. Clinical Benchmark and Translational Threshold

**Figure 3:**
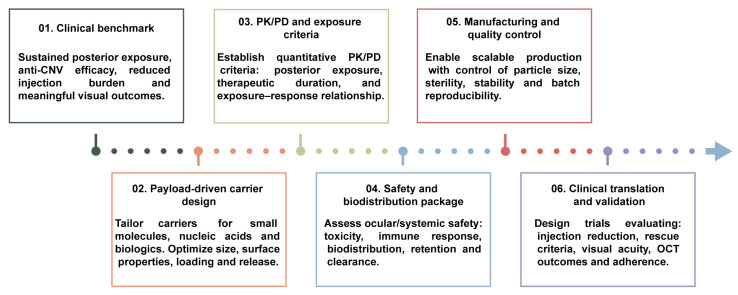
A translational roadmap for developing nanocarrier-based non-invasive ocular delivery strategies for wAMD. This roadmap highlights key developmental stages required for clinical translation, including clinical benchmarking, payload-driven carrier design, quantitative PK/PD evaluation, safety and biodistribution assessment, manufacturing and quality control, and clinical validation. Successful translation requires integration of formulation optimization with posterior segment exposure, exposure–response relationships, long-term safety, scalable manufacturing, and clinically meaningful outcomes, including reduced treatment burden, visual function, and anatomical responses.

Future nanocarrier-based non-invasive ocular delivery should be judged not by the mere detection of drug in posterior tissues, but by its ability to generate sustained, predictable and therapeutically meaningful exposure at relevant targets, including the retina, retinal pigment epithelium (RPE)–choroid complex and CNV lesions. Formulation development should therefore incorporate posterior segment exposure, exposure–response relationships, dosing frequency, long-term safety and patient adherence from the outset (Figure 3).

Intravitreal anti-VEGF therapy remains the clinical standard of care for wAMD [8,9,10,11,12]. Clinically tested topical anti-angiogenic agents, including acrizanib and regorafenib, have not demonstrated sufficient efficacy or durability to support routine use [27,28], underscoring that pharmacological activity and ocular penetration alone are inadequate. Nanocarrier systems must address short precorneal residence, restricted transport across ocular barriers, payload instability and variable posterior segment exposure.

Comparative preclinical studies illustrate both the potential and current limitations of nanocarrier-enabled delivery. In laser-induced CNV mice, topical A-PA-αCPP eye drops delivering aflibercept (10 μg/eye), administered three times for 14 days, produced anti-neovascular effects comparable to a single intravitreal aflibercept injection (0.8 μg/eye), as assessed by FFA, OCT, IB4-stained flat mounts and H&E histology [54]. No obvious ocular or systemic toxicity was observed in the evaluated parameters. However, this comparison was based on different dosing schedules and a short follow-up period, and therefore does not establish clinical equivalence to current intravitreal anti-VEGF regimens. Similarly, in Brown Norway rats, intravitreal injection of solid lipid nanoparticles delivering bevacizumab (5 μg/eye) led to greater reduction in lesion area and lesion depth compared with Avastin^®^ (125 μg/eye) at day 14, as assessed by retinal volume measurements [55]. Nevertheless, pre-laser administration, markedly unmatched doses, and limited functional and long-term safety assessment preclude firm conclusions regarding sustained-release benefit or therapeutic superiority. Overall, current studies support delivery feasibility and short-term biological activity, but robust evidence for superior efficacy, long-term safety, reduced treatment burden, or clinically meaningful advantages over standard intravitreal anti-VEGF therapy remains insufficient.

### 6.2. Payload-Specific Carrier Engineering and Formulation Integration

Carrier design should be tailored to payload properties and therapeutic objectives. Particle size, surface charge, morphology, loading capacity, release kinetics and ligand modification can influence ocular retention, barrier transport and posterior segment tissue distribution (Figure 3) [20,29,37]. Liposomes and polymeric nanoparticles may improve the solubility and retention of hydrophobic small molecules. Nucleic-acid therapeutics require cargo protection, cellular uptake and endosomal escape, whereas anti-VEGF proteins require preservation of structural integrity together with efficient delivery across ocular barriers. Preclinical studies of sunitinib-loaded liposomes, pH-responsive PACD carriers and penetratin/hyaluronic acid-modified liposomes illustrate the value of tailoring carrier design to the therapeutic payload in experimental models of ocular neovascularization [31,56,57].

Nanocarriers may also be integrated with hydrogels or other retention-enhancing systems to extend local residence time and regulate drug release. For example, self-assembled ACD nanoparticles embedded in calcium alginate hydrogel showed sustained anti-angiogenic activity in a primate model with AMD-like features [58]. However, improved ocular retention or tissue penetration does not necessarily ensure adequate drug exposure in the posterior segment or meaningful clinical benefit.

### 6.3. PK/PD, Long-Term Safety, Manufacturability, and Clinical Implementation

Translation will require standardized, quantitative PK/PD frameworks linking posterior segment drug concentrations to durable biological and therapeutic effects. Studies should define exposure–response relationships, duration above therapeutic thresholds and delivery reproducibility in human-relevant large-eye models. Comparative evaluation against approved intravitreal anti-VEGF therapies should ultimately incorporate clinically meaningful outcomes, including rescue-treatment requirements, best-corrected visual acuity, OCT-based anatomical measures and treatment burden.

Safety and product development must proceed in parallel. Priorities include repeat-dose tolerability, ocular surface irritation, retinal toxicity and function, immunogenicity, ocular biodistribution, tissue retention, degradation, clearance and systemic exposure. Scalable manufacture, sterility assurance, long-term stability, batch reproducibility and robust quality control should be embedded early in formulation development [59]. Although approved anterior-segment nanoformulations, such as cyclosporine nanomicellar ophthalmic solution, demonstrate the clinical feasibility of ophthalmic nanomedicine, they do not validate non-invasive posterior segment delivery for wAMD. Clinical translation will require coordinated advances in payload-specific carrier design, PK/PD evaluation, long-term safety assessment, scalable manufacturing and head-to-head comparison with current anti-VEGF therapy (Figure 3).

## 7. Conclusions

Compared with intravitreal injection, nanocarrier-based non-invasive ocular delivery may avoid needle-related procedures and could potentially reduce treatment burden and improve patient acceptability. Nanocarriers such as liposomes, polymeric nanoparticles, and polymeric micelles can improve drug solubility and stability, prolong ocular surface residence, and facilitate tissue penetration, thereby representing a less invasive and potentially safer route for ocular drug administration [19,20]. However, these systems should currently be viewed as investigational or complementary approaches rather than direct replacements for approved intravitreal anti-VEGF therapies.

Nevertheless, whether these systems can achieve sufficient, stable, and safe drug exposure at wAMD lesion sites after non-invasive administration remains the central barrier to clinical translation. Complex ocular barriers continue to restrict drug delivery to the posterior segment [19]. In addition, pharmacokinetic instability, dose–safety trade-offs, formulation consistency, quality control, and manufacturing scale-up further complicate the transition of ophthalmic nanomedicines from laboratory development to clinical application [59]. For macromolecular biologics, including anti-VEGF antibodies and fusion proteins, large molecular size, hydrophilicity, and limited barrier-crossing capacity impose additional technical challenges for non-invasive delivery [18].

Therefore, future nanocarrier-based delivery strategies should prioritize both delivery efficiency and clinical feasibility. Specific priorities include optimization of key formulation parameters, such as particle size, zeta potential, drug loading, release kinetics, and surface modification, as well as protein stability, encapsulation efficiency, and barrier-crossing capacity for biologic payloads. Importantly, regulatory and manufacturing requirements should be considered early in development, rather than only after proof-of-concept efficacy has been demonstrated. Key translational issues include scalable and reproducible production, sterilization, storage conditions, quality-control assays, and compatibility with clinically realistic dosing schedules. Successful commercial translation will also depend on dosing convenience, patient adherence, shelf life, cost, and whether these systems offer a clear clinical advantage over established intravitreal therapies. With continued integration of nanomaterial engineering, ocular pharmacokinetics, and translational medicine, nanocarrier-based non-invasive delivery may hold considerable potential for wAMD treatment. However, further preclinical and clinical studies are warranted to establish its efficacy, safety, and pharmacokinetic profile prior to clinical translation.

## Data Availability

No new data were created or analyzed in this study. Data sharing is not applicable to this article.
